# Profile of Patients with Gallstone Disease in a Sub-Saharan African General Surgery Department: A Retrospective Cohort Study Protocol

**DOI:** 10.29337/ijsp.143

**Published:** 2021-04-27

**Authors:** A. Ndong, N. F. Gaye, J. N. Tendeng, M. L. Diao, A. C. Diallo, F. G. Niang, S. Diop, D. A. Dia, M. Diedhiou, M. Dieng, M. L. Fall, P. M. Ma Nyemb, I. Konaté

**Affiliations:** 1 Department of Surgery, Gaston Berger University of Saint-Louis, Senegal; 2 Department of Imaging, Gaston Berger University of Saint-Louis, Senegal; 3 Department of Anaesthesiology, Gaston Berger University of Saint-Louis, Senegal

**Keywords:** gallstone, lithiasis, biliary tract, surgery, laparoscopy

## Abstract

**Introduction:**

Gallstone disease is a disorder characterised by the formation of stones in the biliary tract. It is the most common biliary condition accounting for more than 98% of all gallbladder and biliary tract disorders. In Africa, previous studies have shown a relative rarity of this condition with a prevalence less than 5%; since it is between 2 and 5 times higher in other continents. A good knowledge of the profile of patient with gallstone disease in a surgical setting could allow to reduce gallstone disease complications and to tailor better the treatment. To our knowledge, there was no previous study about gallstone disease in this region even if there is a high prevalence of metabolic factors of gallstone disease.

**Methods:**

This study objective is to describe the epidemiological, diagnostic and therapeutic profile of patients with gallstone disease at the Department of General Surgery of Saint-Louis Hospital (Senegal). It will be a single-centre retrospective cohort study in a period of 5 years (January 2015 – December 2020). The patients’ record of the department of general surgery will be consulted and the patient contacted if there are missing data. Patients with gallstone disease diagnosed with imaging (ultrasonography and/or CT scan) regardless the presentation (asymptomatic, biliary colic, cholecystitis, common bile duct lithiasis, angio-cholitis, pancreatitis) will be included. Adults and paediatric patients will be enrolled. Patient records lacking sufficient data will be excluded. Studied parameters will be epidemiological, clinical, paraclinical and therapeutic aspects.

**Ethics and dissemination:**

Anonymity and confidentiality of information collected in patients will be respected. This research protocol will be submitted to the Ethics Committee of our institution for approval. The knowledge of the profile of patients with gallstone disease in a surgical setting could allow to reduce gallstone disease complications and to tailor better the treatment. Finally, it will help to reduce the burden of gallstone disease.

**Highlights:**

- Gallstone disease is the most common biliary tract condition
- To our knowledge, there was no previous study about gallstone disease in this region even if there is a high prevalence of risk factors of gallstone disease (sickle cell disease, diabetes, obesity, hypercholesterolemia)
- Good knowledge of the profile of patients with gallstone disease in a surgical setting could allow to reduce gallstone disease complications and to tailor better the treatment.

## 1. Background

Gallstone disease is a disorder characterised by the formation of stones in the biliary tract. It is the most common biliary condition accounting for more than 98% of all gallbladder and biliary tract disorders [1]. Since most stones are asymptomatic for a long time, it is difficult to determine its exact prevalence in the general population. However, autopsy statistics have shown a frequency of 8-9% in adults [2].

More recently with the widespread use of ultrasonography, the global prevalence of gallstones was estimated between 10 to 15% in the general population, with significant variation between countries [3]. In developed countries, this prevalence is higher as in North America, where between 20 and 30 million people suffer from it [3,4]. Therefore, in Africa, previous studies have shown a relative rarity of this condition with a prevalence of less than 5%; since it is between 2 and 5 times in other continents [3,5].

Several etiological factors exist classified according to cholesterol or pigment stones. For pigment cholelithiasis, sickle cell disease is the most common aetiology and its prevalence increases with age and with the severity of haemolysis [6]. It is relatively common in Africa where it remains the first reported risk factor associated with gallstone [7]. Cholesterol stones risk factors are mostly female gender and environmental factors (metabolic syndrome, obesity, high-calorie diet, hypertriglyceridemia) [4]. Along the same lines, type 2 diabetes is associated with an increased risk for gallstone disease [4].

If any treatment is realised, the risk of complication (migration, infection) is important with significant morbidity [8]. Approximately, between 20% and 40% of patients with gallstones will develop gallstone-related complications [9]. The treatment is mainly surgical with cholecystectomy realised preferably by laparoscopy. Approximately 650,000 to 700,000 cholecystectomies are performed each year in the United States [1]. There is a similar trend in Africa where recent studies have shown that the number of cholecystectomies has increased significantly in the last 10 years [10]. In addition, sickle cell patients are operated in a younger age to avoid the occurrence of complication [11].

## 2. Rationale

In Senegal, the literature evaluating the epidemiology of gallstone disease remains scarce. Most of the studies report the treatment of the complications and the results. Studies have shown that laparoscopic cholecystectomy is a common surgery accounting for 21% of all laparoscopic surgeries [12]. There is a high frequency of the main risk factor of gallstone disease which is sickle cell disease. Its prevalence in the general population in Senegal is estimated at 10% [13]. Besides, a study in Dakar (the capital) have shown that 9.4% with sickle cell disease present gallstones [14].

Furthermore, there is a high prevalence of metabolic factors of gallstone disease in the region of Saint-Louis (in the north of the country) where is located our surgical department (diabetes:10.4%, obesity: 25%, hypercholesterolemia 56%) [15,16]. With the increasing prevalence of the metabolic risk factors for gallstone disease such as “Western-type” diet, aging populations, knowing the profile of the patients is crucial [4]. Recognizing modifiable risk factors should help to prevent cholelithiasis. Good knowledge of the profile of patients with gallstone disease in a surgical setting could allow to reduce gallstone disease complications and to tailor better the treatment.

## 3. Methods

The methodology of this protocol is reported in line with the STROCSS guidelines for the reporting of cohort studies in surgery [17]. This protocol has been registered at Research Registry (*https://www.researchregistry.com/*; Number: 6698).

### 3.1. Objective of the study

This study objective is to describe the epidemiological, diagnostic and therapeutic profile of patients with gallstone disease at the Department of General Surgery of Saint-Louis Hospital (Senegal).

### 3.2. Hypotheses

The main hypotheses of this study in our surgical department are:

- Sickle cell disease and metabolic syndrome is the most frequent etiological factor of gallstone disease.
- Ultrasonography is the main imaging modality for the diagnosis of gallstone disease.
- Cholecystitis is the most common complication of gallstone disease.
- Open surgery is more used for the treatment than laparoscopy.

### 3.3. Study setting

Senegal is a West African state considered as a low-income country. Saint-Louis the second city of the country located at 260 km from Dakar the capital. Saint-Louis Regional Hospital is the public referral hospital of the north region of the country and is affiliated with the Faculty of Health Sciences of Gaston Berger University of Saint-Louis since 2010. To our knowledge, there was no previous study about gallstone disease in this region even if there is a high prevalence of metabolic factors of gallstone disease (diabetes: 10.4%, obesity: 25%, hypercholesterolemia: 56%) [15,16].

### 3.4. Type and period of study

It will be a single-centre retrospective cohort study in a period of 5 years (January 2015 – December 2020). The patients’ files (paper records) of the department of general surgery will be consulted and the patient contacted if there are missing data.

### 3.5. Inclusion criteria

Patients with gallstone disease diagnosed with imaging (ultrasonography and/or CT scan) regardless the presentation (asymptomatic, biliary colic, cholecystitis, common bile duct lithiasis, angio-cholitis, pancreatitis) will be included. Adults and paediatric patients will be enrolled. Patient records lacking sufficient data will be excluded. A percentage of loss of follow up between 5 and 20 % will be considered as acceptable [18].

### 3.6. Data analysis

The qualitative variables will be described in number with their proportion and the quantitative variables in the form of mean with their standard deviation.

### 3.7. Data collection and entry

Data collection will be retrospective on a survey form, entered into SPSS 26 software where statistical analyses will be done. Graphs and tables will be made in Excel. The records of the patients realised during consultation or hospitalisation will be used.

### 3.8. Studied parameters

The studied parameters will be:

- Epidemiological data: age, gender, profession;
- Clinical data: presentation (asymptomatic, biliary colic, cholecystitis, common bile duct lithiasis, angio-cholitis, pancreatitis) consultation delay, duration of symptoms, sickle cell disease, existence of metabolic risk factor (obesity, diabetes, dyslipidemia, hypertension), alcohol and tobacco use, contraception, blood pressure, systolic pressure index, temperature, heart rate, respiratory rate, weight, height, body mass index, waist circumference;
- Paraclinical data: hepatic test (SGOT, SGPT, prothrombin time), fasting blood glucose, hemoglobin (anemia), hematocrit (hemoconcentration), white blood cell (leukocytosis), creatinine level, imaging modality (ultrasonography, CT scan), location of the stone (gallbladder, intra hepatic duct, common bile duct, pancreas);
- Therapeutic data: type of treatment (medical treatment, open surgery, laparoscopic surgery, cholecystectomy, choledochotomy with extraction of stones); post-operative course (morbidity, mortality, anatomopathological examination).

### 3.9. Ethics and dissemination

Anonymity and confidentiality of information collected in patient will be respected. This research protocol will be submitted to the Ethics Committee of our institution for approval.

The results of this study will be presented to national and international conferences and published in peer-reviewed journals. Besides, the knowledge of the profile of patients with gallstone disease in a surgical setting could allow to reduce gallstone disease complications and to tailor better the treatment. Finally, it will help to reduce the burden of gallstone disease.

## Registration of research studies

We have registered our study with unique identifying number: researchregistry 6698.
